# Highly Sensitive TiO_2_/Au/Graphene Layer-Based Surface Plasmon Resonance Biosensor for Cancer Detection

**DOI:** 10.3390/bios12080603

**Published:** 2022-08-05

**Authors:** Shahriar Mostufa, Tarik Bin Abdul Akib, Md. Masud Rana, Md. Rabiul Islam

**Affiliations:** 1 Department of Electrical & Electronic Engineering, Rajshahi University of Engineering & Technology, Rajshahi 6204, Bangladesh; 2 School of Electrical, Computer and Telecommfiunications Engineering, University of Wollongong, Wollongong, NSW 2522, Australia

**Keywords:** cancer detection, surface plasmon resonance biosensor, FEM, angular interrogation, biosensor, numerical approach

## Abstract

In this article, a hybrid ${\mathrm{TiO}}_{2}$/Au/graphene layer-based surface plasmon resonance (SPR) sensor with improved sensitivity and capability for cancer detection is presented. The finite element method (FEM) was used for numerical analysis. The proposed SPR biosensor was structured based on the angular analysis of the attenuated total reflection (ATR) method for the detection of various types of cancer using the refractive index component. The resonance angle shifted owing to the increment of normal and cancerous cells’ refractive index, which varied between 1.36 and 1.401 for six different types of normal and cancerous cells. According to numerical results, the obtained sensitivities for skin (basal), cervical (HeLa), adrenal gland (PC12), blood (Jurkat), and breast (MCF-7 and MDA-MB-231) cancer cells were 210 deg/RIU, 245.83 deg/RIU, 264.285 deg/RIU, 285.71 deg/RIU, 292.86 deg/RIU, and 278.57 deg/RIU, respectively. Furthermore, the detection accuracy (DA), figure of merits (FOM), and signal-to-noise ratio (SNR) were also obtained, with values of 0.263 deg^−1^, 48.02 RIU^−1^, and 3.84, respectively. Additionally, the distribution of the electric field and the propagation of the magnetic field for resonant and non-resonant conditions of the proposed structure were illustrated. It was found that an enhanced field was exhibited on the surface of the plasmonic material for resonant conditions. We also measured the penetration depth of 180 nm using decayed electric field intensity. Furthermore, the impact of using a ${\mathrm{TiO}}_{2}$/Au/graphene layer was demonstrated. We further conducted analyses of the effects of the thickness of the gold layer and the effects of additional graphene layers on overall sensitivities for six different types of cancer. The proposed T${\mathrm{iO}}_{2}$/Au/graphene layered structure exhibited the highest overall sensitivity in terms of detecting cancerous cells from healthy cells. Moreover, the proposed sensor was numerically analyzed for a wide range of biological solutions (refractive index 1.33–1.41), and the sensor linearity was calculated with a linear regression coefficient (R^2^) of 0.9858. Finally, numerical results obtained in this manuscript exhibited high sensitivity in comparison with previously reported studies.

## 1. Introduction

Recently, cancer has become one of the main causes of mortality all over the world. Nearly 10 million deaths are caused worldwide due to cancer, according to the World Health Organization (WHO) [1]. The world’s population is estimated to reach approximately 8.3 billion by 2025, of which more than 20 million modern cases of cancer will be reported each year [2]. Cancer is a disease that causes the rapid and uncontrollable creation of abnormal cells, and soon, these abnormalities spread throughout the whole body and damage different body organs, including healthy tissue and valuable organs, finally resulting in death. Due to the leading causes of death being by various types of cancers worldwide, early detection has become essential for diagnosing these diseases [3]. Early detection of cancer means the detection of tumors in the early stages of its development, and it is expected that with this strategy, the process of recovery will advance [4]. According to a UK research group, spotting cancer at an early stage enhances the chance of survival and increases treatment success [5,6]. In the time it takes for signs to become apparent, the expansion of cancer may already have initiated, causing it to be harder to treat. In this respect, a number of biosensor screening tests have been developed for the early detection of cancer, and significant improvements have been made in biosensing applications to detect cancer cells from healthy cells to detect cancer early and reduce mortality [7,8]. 

Biosensor technology is a flourishing field to fulfill the need for sensitive and rapid detection problems [9]. At first, Clark et al. proposed the first biosensor [10] for detecting blood glucose levels. Later, various methods were developed, and insignificant improvements have been shown for biosensor applications. These reported studies could be advantageous in numerous critical applications, for instance, electrochemistry [11,12], immunocytochemistry [13,14], microfluidic devices [15,16], and Raman spectroscopic imaging [17,18]. Recently, numerous microfluidic-based biosensors have been developed. For instance, for the purpose of detecting various refractive index solutions, a hollow silica capillary (HSC) of extremely sensitive refractive index structure has been created as a small, very sensitive optic-fiber SPR sensor implanted in a microfluidic chip [19], which can be used to quantitatively determine biological compounds using a microfluidic chip. The detection of latent membrane protein 1 (LMP1) for an Epstein–Barr virus (EBV) diagnosis was achieved by using a nanofluidic preconcentrated Fano resonance biosensor with low-abundance materials integrated with a nanoslit to the proper concentrations for nanoslit SPR sensing with four distinct methods using the constructed device [20]. Moreover, the DNA sequence of latent membrane protein 1(LMP1) can be detected using a microfluidic polymerase chain reaction (PCR) and a gold nanoslit-based surface plasmon resonance (SPR) sensor. Electrostatic interactions caused the LMP1 DNA probe to be adsorbed onto the integrated device’s SPR chip for additional detection. By amplifying gene fragments at the front end and detecting them at the back end, this all-in-one device can shorten the time needed for analysis without sacrificing accuracy or sensitivity [21]. A localized surface plasmon resonance (LSPR) sensing array and parallel microfluidic channels were combined to create a multiplex detection system, and human immunoglobulin G was used to demonstrate multiplex detection (IgG) and IgM and IgA. These are for the purpose of demonstrating the idea that parallel sensing devices can be utilized to detect various targets, and varying quantities of human IgG, IgA, and IgM were generated [22]. Finally, Celina M. Miyazaki et al. [23] have explained a highly integrated centrifugal lab-on-a-disc (LoaD) platform for automating every step of the process, from plasma extraction to subsequent aliquoting to five parallelized reaction channels for quantitative SPR detection by a low-cost smartphone with a sample to attain automatic SPR detection of targets from bio samples. 

At present, for cancer detection, a number of optical fiber sensors based on surface plasmon resonance (SPR) have been developed due to their portability, compactness, and better sensing performance [24,25,26,27,28]. For instance, Prakhar Dutta et al. [29] demonstrated a centrifuge powered by solar energy that effectively separates blood cells from blood samples using a steady centrifugal force for analyte measurement. Moreover, the peak shift of the resonant spectrum of a nanoplasmonic device (nanoslit SPR chip) through a spectrometer was used. Therefore, surface plasmon resonance is a well-established technique and has shown great potential in various areas such as temperature monitoring, pollution monitoring, chemistry, meteorology, and biomedical applications [25,26]. For biological applications, sensitive and label-free detection is possible with nanostructure-based sensors. Sharp and asymmetric Fano resonances were produced in transmission spectra by a transverse magnetically polarized pulse in these gold nanostructures. A thermal-annealed template-stripping SPR sensor has been shown to be more sensitive to intensity than prior nanoslit and nanohole arrays made using focused ion beam (FIB) and electron-beam lithography (EBL) techniques, according to Kuang-Li Lee et al. [30]. Additionally, the couplings of direct slit transmission or localized surface plasmon resonance (LSPR) in the nanoslits and Bloch wave surface plasmon polariton (BW-SPPs) on the periodic metal surface cause transmission peaks and dips in the spectrum. The interaction of direct slit transmission (a continuous state) and BW-SPPs (a discrete state) results in a Fano resonance profile. The prospect of optical fiber sensors has also been explored for detecting the various cancer types. In [31], Asli et al. demonstrated an SPR-based photonic crystal fiber (PCF) sensor to analyze skin, blood, adrenal gland, breast, and cervical cancer cells in the visible wavelength. Interferometer cascading with a fiber Bragg grating (FBG) [24] was experimentally demonstrated by Sun et al. for detecting breast cancer (HER2) using the refractive index component. Photonic crystal waveguide-based disease detection by Chopra et al. [32] had shown detection of basal, HeLa, and MDA-MB-231 cancer cells using the refractive index change due to cancer using a higher wavelength [32]. Similarly, another work on a photonic crystal platform for cancer detection was done by Sani et al. [33] based on normal and cancer cells with refractive index variation. In 2020, Mollah et al. [34] proposed an early blood cancer detection model. Again, some data regarding cancer cell mapping and refractive index variation can be found in [31,35]. Moreover, for a better understanding of the photodamage in human lung epithelial cancer cells exposed to nanosecond pulses of light, a fractional model was developed, and the laser irradiation of the human cells under study, with light controlled by a Chen chaotic system, was used to monitor variations in energy transference and control optical damage [36]. Finally, Belal et al. [37] proposed a BlueP/MoS_2_ structure-based cancer detection method recently, but the reported sensitivity is only 185 deg/RIU, whereas our proposed sensor exhibits 58% higher angular sensitivity. Although several other optical cancer detections process has been proposed, angular SPR-based detection is a more sensitive and much less complex structure to fabricate.

In recent times, the enormous enhancements in the fabrication technology and the sensitivity of the SPR-based prism-coupled Kretschmann configuration sensors have remarkably improved. Additionally, having a graphene layer on the sensing medium exhibits both excellent conductivity and the ability to stably absorb biomolecules. The main reason behind the absorption of a biomolecule with a graphene layer is the carbon-based rings that widely exist in biomolecules. In addition to having a special molecular structure, the p-stacking interactions between graphene hexagonal cells and carbon-based ring structures are widely present in bio/nanomolecules [38,39]. Due to the bio-functionalization of graphene-based nanomaterials with multiple cells and biomolecules, along with their improved solubility, biocompatibility, and selectivity, graphene and its derivatives exhibit some interesting applications in mass spectroscopy, optical and electrochemical sensors and electronic device bio-imaging [40,41,42,43,44]. A precondition for such a diagnosis is the evaluation of cancer biomarkers. A graphene-based electrochemical array with a number of functionalized molecules acting as probes is used to construct sensors for circulating tumor cells. Indeed, drug-resistant cells, circulating tumor cells, and other cancer cells may all be detected using graphene sensors. According to their varying affinities, seven distinct types of molecules may be fixed onto the graphene surface to serve as probes for differentiating different cell types [45]. This sensor array makes contact with different cell surfaces, and a corresponding high-affinity probe seizes the target cells. Such a perfect biosensor can distinguish between cells at the single-cell level, and various concentrations elicit different reactions. As a result, the sensor has outstanding performance and can identify cells with great sensitivity. The graphene array-based electrochemical biosensor exhibits outstanding accuracy, great sensitivity (lowest detectable limit: one cell), and superior stability for cell detection and precise cancer diagnosis [45]. Therefore, employing a sensing medium on the surface of the graphene layer would improve the diversity of biosensing applications as well as enhance the biological detection capability. As a result, the proposed biosensor in this paper has been designed employing such layers. 

In this paper, the main aims are to determine the refractive index changes in cancer cells for early cancer detection purposes. For this reason, the design and analysis of a BK_7_/${\mathrm{TiO}}_{2}$/Au/graphene-based SPR sensor model is proposed for detecting skin (basal), cervical (HeLa), adrenal gland (PC12), blood (Jurkat), and breast (MCF-7 and MDA-MB-231) cancer cells using the refractive index component. The novelty of the proposed work is that proper FEM-based numerical approaches are used to propose a model of a highly sensitive hybrid SPR-based biosensor for the detection of cancerous cells from healthy cells, and the resultant sensitivity of our proposed sensor is also compared with some recently reported SPR-based biosensors. Moreover, a demonstration of the practical fabrication process, the impact of material layers, an analysis of the gold layer thickness, an analysis of the graphene layer, and a wide range of biological solution detections with sensor linearity measurements are also presented. The numerical results demonstrate that the proposed sensor exhibits sensitivity in the detection of skin (basal), cervical (HeLa), adrenal gland (PC12), blood (Jurkat), and breast (MCF-7 and MDA-MB-231) cancer cells, with values of 210 deg/RIU, 245.83 deg/RIU, 264.285 deg/RIU, 285.71 deg/RIU, 292.86 deg/ RIU, 278.57 deg/RIU, respectively. Furthermore, the detection accuracy (DA), figure of merits (FOM), and signal-to-noise ratio (SNR) are also obtained, with values of 0.263 deg^−1^, 48.02 RIU^−1^, and 3.84, respectively.

## 2. Design Methodology

### 2.1. Sensor Structural Design

**Figure 1:**
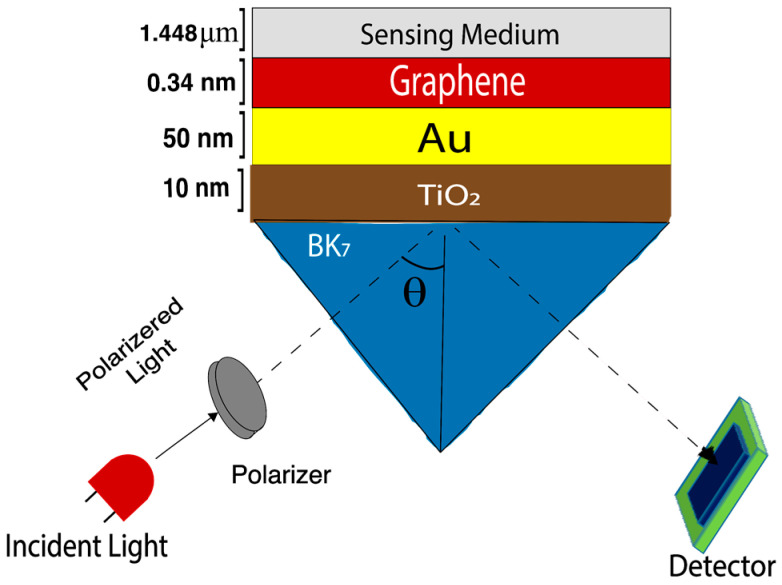
Structural arrangement for ${\mathrm{BK}}_{7}$/${\mathrm{TiO}}_{2}$ /Au/graphene-based heterostructure biosensor.

The proposed ${\mathrm{BK}}_{7}$/${\mathrm{TiO}}_{2}$/Au/graphene-based heterostructure biosensor works on the basis of the Kretschmann configuration [46]. The following sensor is illustrated in Figure 1 and was designed for a monochromatic wavelength (λ) of 633 nm (He-Ne laser) [47]. Table 1 illustrates the material refractive index properties as well as each layer thickness used in the proposed sensor modeling. A p-polarized © light having a wavelength (λ) of 633 nm was incident at an acceptance angle on a prism (${\mathrm{BK}}_{7}$), and the angular interrogation technique was applied to determine the resonance angle. As the incident light wave passes through multiple layer interfaces, reflection intensity decays at the output, and in the resonance condition, the output reflectance intensity achieves its minimum value. The output reflectance intensity can be monitored using a charge-coupled device (CCD) or complementary metal-oxide (CMOS) [47]. For calculating the prism $({\mathrm{BK}}_{7}$) refractive index for incident wavelength (λ) Equation (1) has been used, as follows [46].

(1) $$n_{\mathrm{Bk}7}=\sqrt{1+\frac{1.03961212\lambda^{2}}{\lambda^{2}-0.00600069867}+\frac{1.01046945\lambda^{2}}{\lambda^{2}-103.560653}+\frac{0.231792344\lambda^{2}}{\lambda^{2}-0.0200179144}}$$

For the second layer, titanium dioxide (TiO_2_) was used as an adhesion thin film to provide strong interaction between the prism and plasmonic metal layer and enhance the refractive index sensitivity due to its high refractive index and good chemical stability [48,49,50,51]. Gold (Au) material was used as a plasmonic material on the third layer due to its larger resonance angle shifts for the dielectric medium’s refractive index change. In addition to that, gold (Au) is also a chemically highly stable material [41,52]. The refractive index of the gold (Au) layer was obtained from Equation (2), known as the Drude–Lorentz model [53] for an incident wavelength (denoted as λ) of 633 nm, where the collision wavelength ($\lambda_{c}$) is $8.9342\;\times 10^{-6}\;$m and the plasma wavelength ($\lambda_{p})$ is $1.6826\;\times 10^{-7}$m [53].

(2) $$n_{\mathrm{Au}}=\sqrt{\left(1-\frac{\lambda^{2}\times \lambda_{c}}{\lambda_{p}^{2}\left(\lambda_{c}+\lambda \times i\right)}\right)}$$

The refractive index of graphene for the incident wavelength (λ) was calculated using Equation (3). Graphene exhibits the best optomechanical and optoelectronic properties of high confinement, low loss, vast surface-to-volume ratio, and turnability. Due to having carbon atoms organized in hexagonal shapes, graphene ensures good interactions with the biological sample molecules or analytes. Again, the enhancement of the electric field at the nano interface occurs due to the enhanced coupling when graphene is introduced to the metallic films [41,42,43,44]. Furthermore, among 2D materials, graphene has versatile biocompatibility and more remarkable absorption abilities [56].

(3) $$3+\frac{i\times C\lambda }{3}$$

where C is 5.446 ${\mu m}^{-1}$ and the mono graphene layer thickness is 0.34 nm [46,54,55]. Finally, the sensing medium layer marked in Figure 1 was used as an analyte or sample placing region for the proposed SPR sensor. Depending on the variation of biological samples, the analyte optical (the refractive index properties) of the sensing medium were varied according to the skin (Basal), cervical (HeLa), adrenal gland (PC12), blood (Jurkat), and breast (MCF-7 and MDA-MB-231) healthy and cancer cells’ refractive index variations. The data of the analyte’s refractive index in Table 2 was obtained from a recently reported work [31,57,58]. The normal cancer cell size varies depending on the type of cancer cell. For instance, the cervical cancer cell is 17.66 μm, the basal cancer cell is 30 μm, and the breast cancer cell is 17.48 or 18.72 μm [59]. Although the size of cancer varies depending upon the region of the human body, the sensing mechanism of cancer cells is in the form of liquid biopsy or infiltration blood fluid samples, and it requires a very minimum quantity [59]. Liquid biopsy is a minimally invasive method that uses samples of blood, cerebrospinal fluid, urine, sputum, ascites, and, in theory, any other bodily fluid. It is gradually emerging as a practical substitute for monitoring cancer patients in real-time and evaluating biomarkers that are often only examined in tissue biopsies [60,61,62,63]. 

### 2.2. Mathematical Modeling 

The p-polarized or TM-polarized light reflectance intensity measurement is crucial for the sensing purpose of the SPR sensor. Surface plasmon waves (SPWs) are transverse waves with an oscillating electric field normal to the surface. The transverse magnetic polarization TM state describes how the surface plasmon spreads as an electromagnetic wave parallel to the x direction with a magnetic field orientated parallel to the y direction. Since surface plasmons only have an electric field component, which is normal to the surface, therefore, the initial prerequisite for SP excitation is the condition of the TM polarization state, which is required to produce the distribution of charges on the metal contact and to satisfy the boundary conditions necessary to excite SPR detailed in [64,65]. The reflectance intensity of the proposed sensor can be expressed as follows [66,67]:

(4) $$R_{p}=\left|r_{p}^{2}\right|\cdot$$

(5) $$r_{p}=\frac{\left(M_{11}+M_{12}q_{N}\right)n_{1}-\left(M_{21}+M_{22}q_{N}\right)}{\left(M_{11}+M_{12}q_{N}\right)n_{1}+\left(M_{21}+M_{22}q_{N}\right)}$$

Here, $r_{p}$ represents the reflection coefficient for TM-polarized incident light wave, as the proposed sensor is a multilayered structure. Therefore, for a multilayer structure, the transfer matrix function, Mij, is given as follows [66,67]:

(6) $$M_{\mathrm{ij}}=\left(\Pi_{k=2}^{N-1}M_{k}\right)=\left(\begin{array}{cc} M_{11} & M_{12}\\ M_{21} & M_{22} \end{array}\right)$$

where

(7) $$M_{k}=\left[\begin{array}{cc} \cos\beta_{k} & -\left(i\sin\beta_{k}\right)/q_{k}\\ -{\mathrm{iq}}_{k}\sin\beta_{k} & \cos\beta_{k} \end{array}\right]$$

(8) $$q_{k}={\left(\frac{\mu_{k}}{\varepsilon_{k}}\right)}^{1/2}$$

(9) $${\mathrm{Cos}\theta }_{k}=\frac{{\left(\varepsilon_{k}-n^{2}\sin^{2}\theta_{1}\right)}^{1/2}}{\varepsilon_{k}}$$

(10) $$\beta_{k}=\frac{2{\pi d}_{k}}{\lambda }{\left(\varepsilon_{k}-n^{2}\sin^{2}\theta_{1}\right)}^{1/2}$$

Here, the arbitrary phase constant is $\beta_{k}$, and ${\;\theta }_{k}$ is the angle of the entrance for the $k^{\mathrm{th}}$ layer. Again, for the $k^{\mathrm{th}}\;$layer, the thickness and dielectric constant are denoted as $d_{k}$ and $\varepsilon_{k}$, respectively. Furthermore, as the refractive index increases in the analyte medium, the SPR angle shifts right, increasing the output reflection intensity. The following phenomenon is explained using Equation (11), which shows the relationship of the SPR angle and analyte as follows [54,66,68]:

(11) $$\theta_{\mathrm{spr}}=\sin^{-1}\frac{\eta_{\mathrm{eff}\;}\eta_{a}}{\eta_{p}\sqrt{\eta_{\mathrm{eff}}^{2}+}\eta_{a}^{2}}$$

Furthermore, some crucial sensing parameters in the sensing application include the angular shift sensitivity (S), the figure of merits (FOM), and detection accuracy (DA) [66]. The proposed sensor sensitivity was calculated using Equations (12)–(14), where the ${\Delta \theta }_{\mathrm{spr}}$ is the SPR angle or resonance angle change, and Δn is the refractive index variation. The full-width half maxima (FWHM) define 50% of the reflectance curve spectral width. The performance of the sensor measuring formulas is as follows [66]:

(12) $$S=\frac{{\Delta \theta }_{\mathrm{spr}}}{\Delta n}\;[\deg/\mathrm{RIU}]\;$$

(13) $$\mathrm{DA}=\frac{1}{\mathrm{FWHM}}\;[1/\deg]$$

(14) $$\mathrm{FOM}=\frac{S}{\mathrm{FWHM}}\;[1/\mathrm{RIU}]\;$$

Furthermore, the signal-to-noise ratio (SNR) of the real SPR sensing system critically depends on how well one measures the signals with real instruments. It was calculated as follows [47,69]:

(15) $$\mathrm{SNR}=\frac{{\Delta \theta }_{\mathrm{SPR}}}{\mathrm{FWHM}}$$

Finally, using Equations (13)–(15), the overall sensor parameters of detection accuracy (DA), the figure of merits (FOM), and signal-to-noise ratio (SNR) were found to be 0.263 deg^−1^, 48.02 RIU^−1^, and 3.84, respectively, and the full-width half maxima (FWHM) was found to be 3.8 deg. 

### 2.3. Numerical Modeling

**Figure 2:**
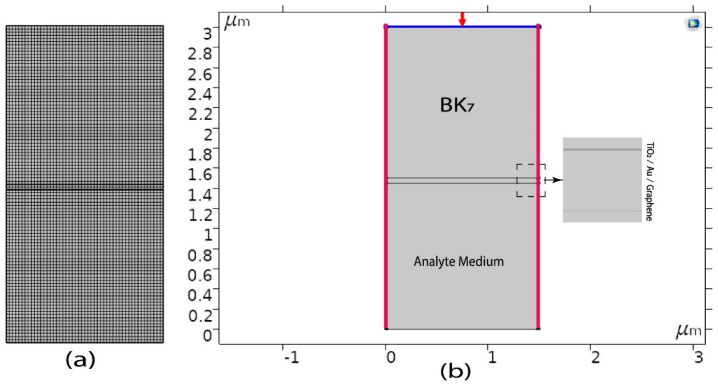
General COMSOL Multiphysics view structure of the proposed (${\mathrm{BK}}_{7}$/${\mathrm{TiO}}_{2}$ /Au/graphene) SPR biosensor. (**a**) Computational meshing domain. (**b**) Numerical simulation model of the proposed structure.

**Figure 3:**
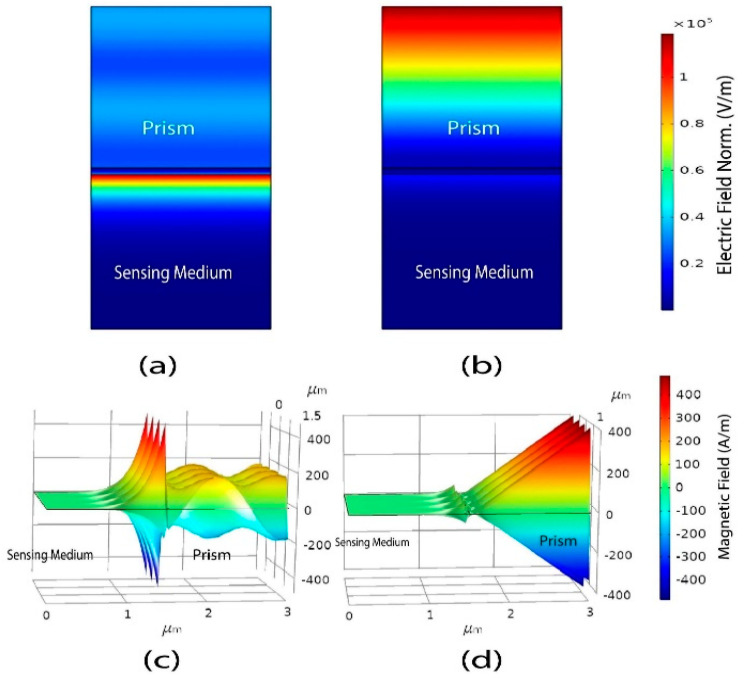
The electric field norm distribution and magnetic field propagation for the proposed hybrid (${\mathrm{BK}}_{7}$/${\mathrm{TiO}}_{2}$ /Au/graphene) SPR biosensor structure at an analyte refractive index of 1.36. (**a**) Electric field distribution at a resonance angle of 75.6 deg exhibiting an enhanced field on the surface of gold (Au). (**b**) Electric field distribution at a non-resonance angle of 88 deg with no enhanced field on the surface of gold (Au). (**c**) A 3D representation of the propagation of the magnetic field’s z component (A/m) at a resonance angle of 75.6 deg. (**d**) A 3D representation of the propagation of the magnetic field’s z component (A/m) at a non-resonance angle of 88 deg.

The design and analysis of the proposed model demonstrated in this paper is a finite element method (FEM)-based numerical simulation. To simulate the proposed model, COMSOL Multiphysics version 5.5 was utilized, and we simulated the 2D geometry of the proposed sensor. The structure of the proposed (${\mathrm{BK}}_{7}$/${\mathrm{TiO}}_{2}$/Au/graphene) SPR biosensor is illustrated in Figure 2b, which shows a light source having a wavelength of 633 nm incident on the top of prism BK_7_. Again, the Floquent periodicity periodic boundary conditions (marked in Figure 2 with red color) and periodic port conditions were applied. For this FEM model, the extremely fine physics-controlled sized mapped mesh having a minimum element size of 6×10^−5^ μm and a maximum element size of 0.03 μm were selected, as illustrated in Figure 2a. Furthermore, to perform the angular interrogation technique, we varied the incident angle of the source, selecting the parametric sweep operation, where the incident angle was simulated for 60 to 89 deg with 0.1 deg incremental deviation. The reflectance intensity was calculated for each incident angle to detect the resonance angle, and by observing the minimum reflectance intensity at the output, we identified the resonance angle from the output intensity curve. The frequency-domain solver was selected to solve the model using a frequency of $3\times 10^{8}/\;\lambda \;$Hz. Finally, by observing the shift in the output reflection intensity curve for the analyte layer refractive index variation, the sensor performance and sensitivity were calculated. In addition to that, to compare the proposed model in SPR and non-SPR conditions, we also demonstrated the electric field intensity and magnetic field propagation both at the resonance and non-resonance angle, which is illustrated in Figure 3. In the resonance condition, due to the strong localization and the maximum excitations of surface plasmons in the plasmonic layer, the electric field and magnetic field are enhanced [54]. In Figure 3a,b, the enhanced electric field intensity can be found on the plasmonic gold layer under resonance conditions, whereas in non-resonance conditions, no electric field intensity on the plasmonic layer was observed. Similarly, for the 3D magnetic field propagation of the z component (A/m), a strong excitation was observed at a resonance angle and at a non-resonance angle. No excitation on the plasmonic layer was detected. Some similar FEM models for simulating multiple layers were also demonstrated in [46,66,70].

**Figure 4:**
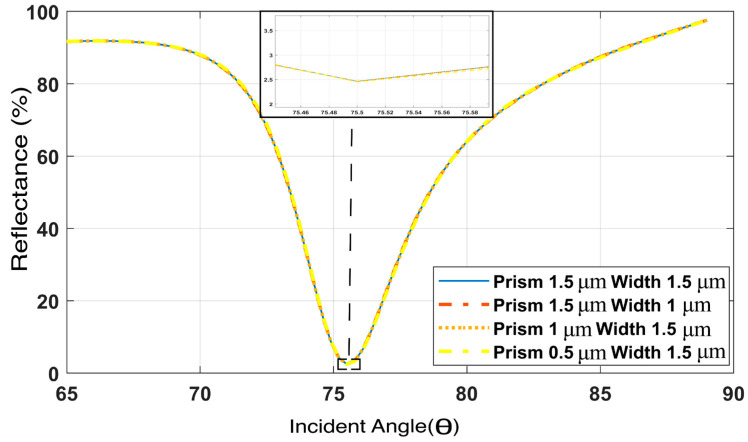
Effect of the height of the prism layer and the total width on the proposed sensor.

Finally, to demonstrate that our proposed numerical sensor area and resonance conditions work properly, we analyzed the prism layer thickness for values of 0.5 μm, 1 μm, and 1.5 μm. As the angular resonance condition does not depend on the prism height, we therefore cannot find any change in the resonance angle due to the changes in prism layer thickness, as illustrated in Figure 4. Again, the layers of ${\mathrm{TiO}}_{2}$/Au/graphene have a particular thickness, as shown above. Therefore, we used a similar ${\mathrm{TiO}}_{2}$/Au/graphene thickness layer.

Again, in terms of width, we used periodic boundary conditions (PBCs). Periodic boundary conditions (PBCs) are a set of boundary conditions that are often chosen for approximating a large (infinite) system by using a small part called a unit cell. PBCs are often used in computer simulations and mathematical models. Therefore, no effect of changing the width of the sensor was found; this was also simulated for widths of 1 and 1.5 μm. Finally, the analyte layer thickness depends on the analyte samples and the penetration depth calculated later in this section for this SPR sensor. Furthermore, the authors of [71] demonstrated that the transfer matrix method (TMM) and the finite element analysis, which exhibit exactly identical results, and the FEM-simulating structure is also appropriate for the study of all TMM-based SPR sensors. 

### 2.4. Electric Field Analysis and Penetration Depth Calculation 

**Figure 5:**
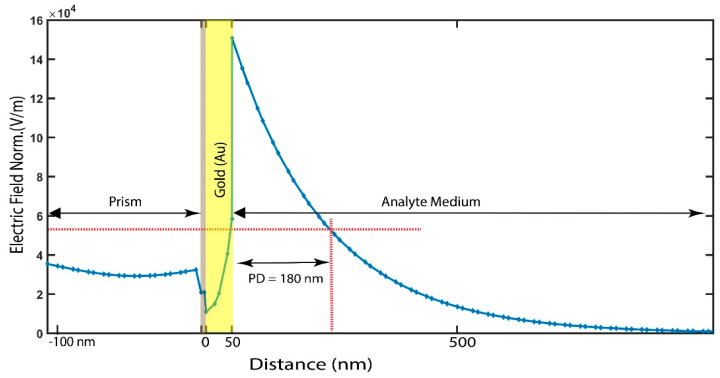
Cross-section plot of the total electric field along the direction perpendicular to the prism base at an analyte refractive index of 1.36 and a resonance angle of 75.6, showing a clear evanescent field at the sensing interface.

To further confirm the strong SPR excitation of the proposed ${\mathrm{BK}}_{7}$/${\mathrm{TiO}}_{2}$/Au (50 nm)/graphene sensor, we employed the electric field distribution of the structure at a resonance angle of 75.6 deg and at analyte 1.36 in Figure 5. As can be seen, a significant electric field augmentation is produced at the sensing surface, and the intensity of the electric field exponentially decreases to the sensing medium, which contains the target biomolecules. The calculated PD for the proposed ${\mathrm{BK}}_{7}$/${\mathrm{TiO}}_{2}$/Au (50 nm)/graphene is 180 nm, which signifies that the interaction volume of the field in the sensing medium is larger. The PD is defined as the distance traveled by the field normal to the layer in the sensing medium, at which the field intensity decays to 1/e (37%) [72,73]. Similarly, another recently reported graphene–MoS_2_-based structure exhibits a PD of 150 nm [73]. Thus, the electric probing field close to the graphene layer is very intense and highly sensitive to biomolecule interactions when using our proposed sensor. 

### 2.5. Practical Fabrication Process

**Figure 6:**
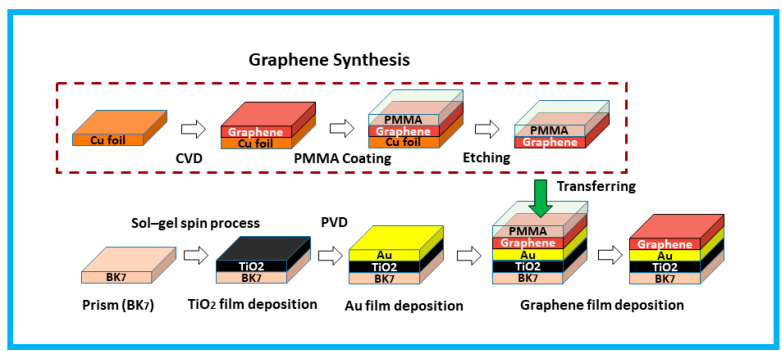
The proposed practical fabrication steps of the BK_7_/TiO_2_/Au/graphene structured-based biosensor.

This paper proposed and analyzed a hybrid ${\mathrm{BK}}_{7}$/$\;{\mathrm{TiO}}_{2}$/Au/graphene-based biosensor with a numerical modeling method, but the practical fabrication of the proposed sensor can also be possible. The practical fabrication steps regarding the proposed sensor are illustrated in Figure 6. Firstly, to fabricate the proposed sensor, Bk_7_ must be chosen as a substrate, and the sol-gel spin method would be used to deposit a thin layer of TiO_2_ on the prism’s base. Isopropyl titanate and isopropyl alcohol would be combined to create the solution for this usage [74,75]. Then, a gold (Au) layer would be deposited on the top of BK7 using physical vapor deposition (PVD) or sputtering techniques. The thickness of the gold layer would depend on the particle sputtering deposition time [66]. In a chamber pressure of less than 1000 °C and at 3.6 Torr, a high-quality graphene film would be deposited on the Copper (Cu) film utilizing the CVD process, where methane (CH_4_) gas would be used as a carbon source [76,77,78,79,80,81]. The PMMA would be used for transferring the graphene deposited layer onto the substrate, and during the 25 °C etching process with sulfuric acid (H_2_SO_4_), the Cu foil would have to be removed. After the transfer of the graphene film by applying acetone ((CH_3_)_2_CO), the PMMA layer would be removed [47].

## 3. Material Impacts on the Proposed Biosensor

### 3.1. Impact of Material Layers on Sensitivity

**Figure 7:**
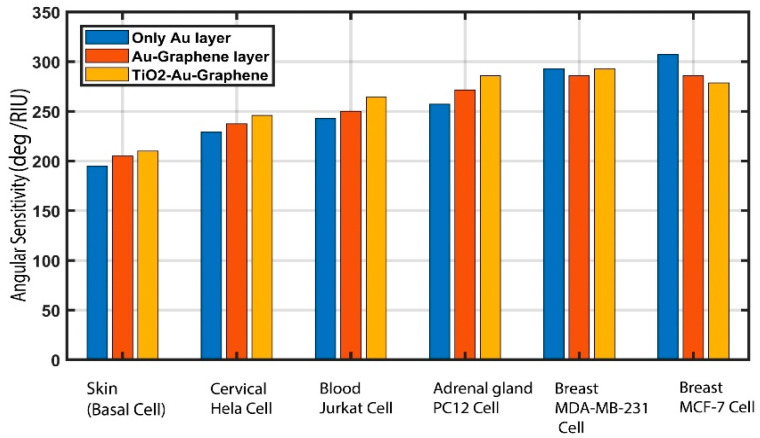
Influence of material layers on angular sensitivity for the following structures: (i) BK_7_/Au/analyte; (ii) BK_7_/Au/graphene/analyte; and the proposed (iii) BK_7_/TiO_2_/Au/Graphene/analyte on the angular sensitivity for various types of cancer detection.

In this section, the significance and impact of each deposited material layer for the detection of cancer are demonstrated. The sensitivity comparison of the influence of different layers is outlined in Figure 7, and the angular sensitivity data are tabulated in Table 3. Here, for the impact of using only the Au layer, a simulation was conducted for the BK_7_/Au/analyte structure. The simulation results demonstrated that, when using only the Au layer, the angular sensitivity of the detection of skin (basal), cervical (HeLa), adrenal gland (PC12), and blood (Jurkat) cancer cells remains much lower compared to the proposed biosensor structure. 

Similarly, to assess the impact of adding graphene, a simulation was undertaken for the BK_7_/Au/graphene/analyte structure. The results demonstrate that, when using graphene with Au, the sensitivity of the detection of skin (basal), cervical (HeLa), adrenal gland (PC12), and blood (Jurkat) cancer cells increased compared to the results of only the Au layer. However, using only graphene with gold (Au) does not exhibit values as high as the proposed biosensor structure. Therefore, applying the TiO_2_ underneath Au enhances the sensitivity of detection of skin (basal), cervical (HeLa), adrenal gland (PC12), and blood (Jurkat) cancer cells. However, the proposed sensor exhibits less sensitivity for breast (MCF-7) cancer cell detection compared with only Au and Au/graphene layers. However, the proposed BK_7_/TiO_2_/Au/graphene/analyte structure exhibits overall high sensitivity for all cancer cell type detection compared to only Au and Au/Graphene structures. Hence, the proposed BK_7_/TiO_2_/Au/graphene/analyte biosensor structure is used in this paper for the various types of cancer detection due to its high overall sensitivity for each cancer type.

### 3.2. Gold (Au) Thickness Effect on Sensitivity

**Figure 8:**
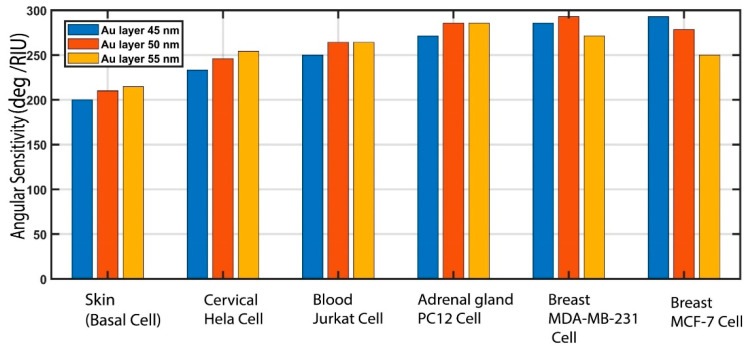
Analysis of the thickness of the gold (Au) layer for 45 nm, 50 nm, and 55 nm in the proposed BK_7_/TiO_2_/Au/graphene biosensor.

In this paper, we propose a biosensor structure of BK_7_/TiO_2_/Au/graphene for cancer detection, where the thickness of the gold layer is taken to be 50 nm. In this section, we demonstrate the effects of the 50 nm gold layer on the various types of cancer detection. To verify the influence of the 50 nm thick gold layer, we varied the gold layer thickness, using thicknesses of 45 nm, 50 nm, and 55 nm within the proposed structure. The results of the angular sensitivity change regarding the assessment of Au layer thickness are tabulated in Table 4 and outlined in Figure 8. From the numerical analysis of these results, it can be seen when comparing an Au layer of a thickness of 50 nm with 45 nm that the angular sensitivity of 50 nm Au is higher for skin (basal), cervical (HeLa), adrenal gland (PC12), blood (Jurkat) and breast (MCF-7 and MDA-MB-231) cancer detection. Similarly, for a 55 nm Au layer thickness, higher sensitivity is initially exhibited for skin (basal) and cervical (HeLa) cancer detection compared to a 50 nm Au layer thickness, but for breast (MCF-7 and MDA-MB-231) cancer cell detection, the angular sensitivity drastically decreases. Therefore, the gold layer thickness was selected to be 50 nm, as this thickness exhibits an overall higher sensitivity for each type of cancer detection. In addition to the numerical analysis of the effect of the thickness of the gold layer on angular sensitivity, it can also be explained analytically. Some reflectance profile parameters are influenced by the characteristics of the metal layer thickness, and the angular sensitivity (S) of the Kretschmann configuration is one of them, which can be explained as follows[64]:

(16) $$S=\frac{\varepsilon_{\mathrm{mr}}\sqrt{-\varepsilon_{\mathrm{mr}}}}{\left(\varepsilon_{\mathrm{mr}}+n_{a}^{2}\right)\sqrt{\varepsilon_{\mathrm{mr}}\left(n_{a}^{2}-n_{p}^{2}\right)-n_{a}^{2}n_{p}^{2}}}$$

Here, n_p_ is the prism refractive index; the real part of the metal-dielectric constant is represented by $\varepsilon_{\mathrm{mr}}$, and the analyte refractive index is represented by n_a_.

### 3.3. Effect of the Graphene Layer on Sensitivity

**Figure 9:**
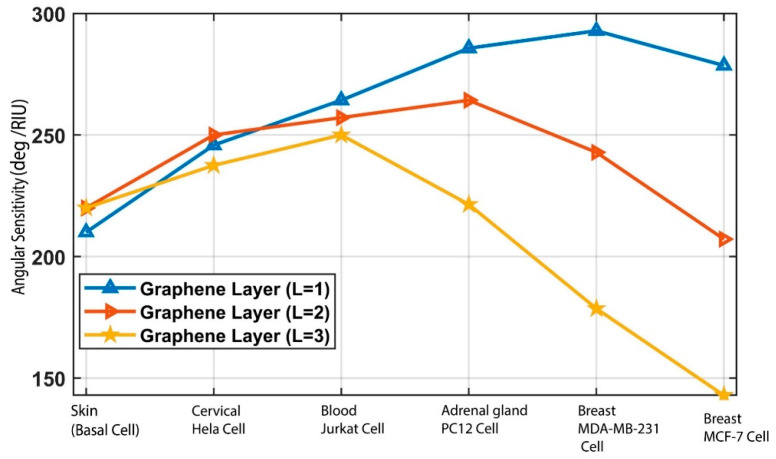
Analysis of the graphene layer for the proposed BK_7_/TiO_2_/Au/graphene biosensor.

Each graphene layer sheet thickness can be denoted as 0.34 × L nm, where (L) represents the graphene layer number [82]. An analysis of the graphene layer was conducted for values of L of 1, 2, and 3 and is shown in Figure 9. The angular sensitivity data are shown in Table 5. For the L = 2 layer, the obtained sensitivities for skin (basal), cervical (HeLa), adrenal gland (PC12), blood (Jurkat), and breast (MCF-7 and MDA-MB-231) cancer cells are 220 deg/RIU, 250 deg/RIU, 257.14 deg/RIU, 264.28 deg/RIU, 242.857 deg/RIU, and 207.14 deg/RIU, respectively. The obtained sensitivities for these cell types for the L = 3 layer are 220 deg/RIU, 237.5 deg/RIU, 250 deg/RIU, 221.42 deg/RIU, 178.5714 deg/RIU, and 142.8571 deg/RIU, respectively. By observing the results, it can be seen that the graphene monolayer L = 1 exhibits better overall sensitivity in comparison with L = 2 and 3. Therefore, we have used a monolayer of graphene in the proposed sensor. 

## 4. Results and Analysis (Cancer Detection)

**Figure 10:**
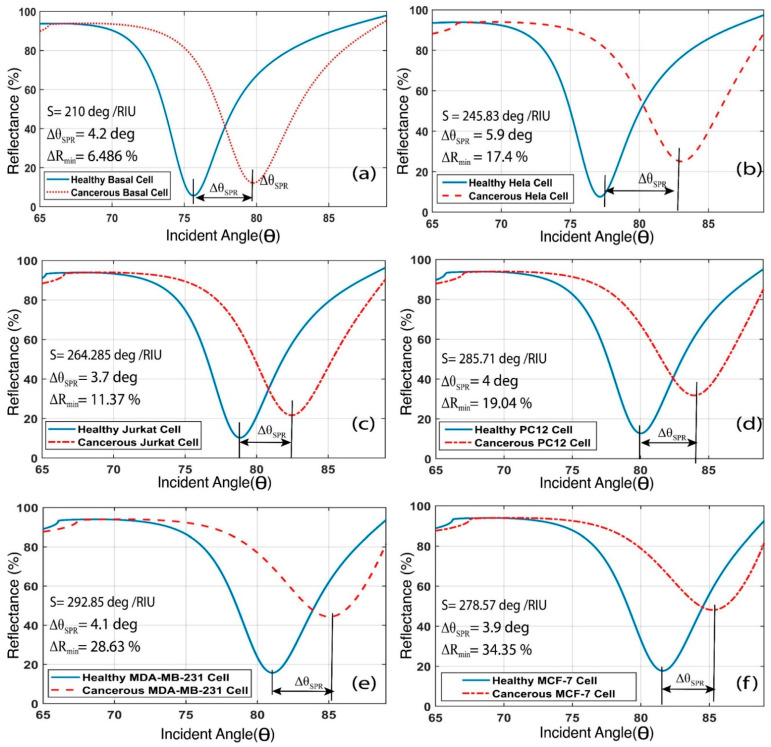
The output reflectance intensity curve with respect to the incident angle and the resonance angle shift due to the refractive index increment for (**a**) basal, (**b**) HeLa, (**c**) Jurkat, (**d**) PC12, (**e**) MDA-MB-231, and (**f**) MCF-7 healthy and cancerous cell analytes.

The method of detecting cancer with the proposed BK_7_/TiO_2_/Au/graphene/analyte structured biosensor is demonstrated in this section. When a normal cell is affected by cancer, the refractive index of the cell increases. The refractive index data for the respective cell types are tabulated in Table 2. These skin (basal), cervical (HeLa), adrenal gland (PC12), blood (Jurkat), and breast (MCF-7 and MDA-MB-231) normal and cancerous cell refractive index data have been used as an analyte layer refractive index to identify the cancerous cells from the normal cells. The output reflectance intensity curves for basal, HeLa, Jurkat, PC12, MDA-MB-231, and MCF-7 cells are illustrated in Figure 10. From the illustrated Figure 10, it can be seen that, due to the refractive index increment between each normal and cancerous cell, the resonance angle shifts rightwards. In Table 6, the resonance or SPR angle for each particular normal and cancerous cell is denoted by $\theta_{\mathrm{SPR}}$. 

Furthermore, for skin (basal), cervical (HeLa), adrenal gland (PC12), blood (Jurkat), and breast (MCF-7 and MDA-MB-231) normal and cancerous cells, the resonance and SPR angle shifts are 4.2 deg, 5.9 deg, 3.7 deg, 4 deg, 4.1 deg, and 3.9 deg, respectively. Therefore, the resonance angle angular shift sensitivity of the skin (basal), cervical (HeLa), adrenal gland (PC12), blood (Jurkat), and breast (MCF-7 and MDA-MB-231) normal and cancerous cells are 210 deg/RIU, 245.83 deg/RIU, 264.285 deg/RIU, 285.71 deg/RIU, 292.86 deg/RIU, and 278.57 deg/RIU, respectively, as calculated using Equation (12). 

## 5. Wide Range of Biological Solution Detections and Sensor Linearity 

**Figure 11:**
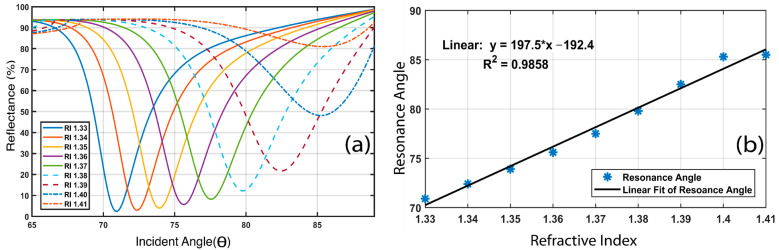
(**a**) The effect of variations in the sensing medium refractive index (RI) on reflectivity and resonance angle for the proposed sensor. (**b**) Resonance angle shift concerning sensing medium refractive index increments and the linear curve fitting where resonance angle is a function of analyte RI.

Although the proposed (BK_7_/TiO_2_/Au (50 nm)/graphene sensor was designed focusing on the various types of cancer detection, it is also capable of detecting a wide range of biological solutions. Most biological solutions have a refractive index in the range of 1.33 to 1.41. Therefore, the numerical results regarding changes in the sensing medium’s refractive index are plotted in Figure 11a. It can be seen by observing the obtained results that the proposed sensor is also capable of detecting a wide range of biological solutions with a resonance angle shift.

In order to measure a high refractive index, sensor linearity is a precondition [46,83]. The sensor linearity from the slope of the linear fitting curve with respect to the resonance angle was measured for the proposed sensor using MATLAB curve fitting. If the sensor shows linearity, then it is easier to predict the resonance angle for a higher analyte refractive index. Again, sensor nonlinearity causes critical variance and makes the detection procedure increasingly intricate. Therefore, nonlinearity is not a desired quality in a sensor. The linearity is depicted using the correlation coefficient (R), and by performing linear regression, this correlation coefficient (R) is acquired. The regression equation for the linear fit of the proposed BK_7_/TiO_2_/Au (50 nm)/graphene sensor is y = 197.5 × x − 192.4, and the regression coefficient is R^2^ = 0.9858, as shown in Figure 11b. It is evident that the value of the correlation coefficient is very close to 1, which indicates linearity close to the ideal. 

Finally, the comparison between our proposed biosensor and some recently reported works demonstrates that our proposed biosensor is highly sensitive in terms of angular sensitivity, DA, FOM, and SNR, as tabulated in Table 7. 

## 6. Discussions

In this article, a surface plasmon resonance-based biosensor has been proposed on the basis of the angular-based detection method for detecting cancer biomarkers. However, there are various methods of detecting biomarkers using the SPR-based method, including the wavelength-based detection method and the reflection intensity-based detection method. Each of these methods detects biomarkers with different approaches, and each has a specific focus on the detection sensitivity. For instance, the spectral mode analysis method can be used to improve the spectral sensitivity and FOM, as demonstrated in [90], but it requires higher wavelengths of 850 nm to 1059 nm, whereas we have used the visible wavelength. Again, a study [91] focused on penetration depth parameter enhancement using wavelength analysis of the surface interactions and analysis of larger biomolecules, such as bacteria cells with a typical size of near 1 μm, whereas, in our paper, we utilized a different method called liquid biopsy as a biomarker for our samples versus directly detecting the whole cell. Furthermore, the demonstration of imaging sensitivity used for a long-range SPR sensor detects biomarkers based on changes in the reflection intensity; therefore, it is capable of detecting very minor changes in the refractive index. Several methods of detecting biomarkers using SPR-based technology are tabulated in Table 8.

## 7. Conclusions

The rapid advancements in biomedical research over the past few years have shown a significant demand for biosensing with high sensitivity, specificity, and throughput. Biosensors with extremely high sensitivity and excellent identification specificity are anticipated to detect certain biomolecules. Surface plasmon sensors are among the best current sensing technologies because of their beneficial qualities, including their high sensitivity, short response times, and ability to conduct real-time label-free sensing when biomolecules interact with the sensor surface. This article proposes a multilayer coated SPR sensor based on graphene for early-stage cancer detection using numerical approaches. Due to the fact that the design and characteristics of multilayer arrangements substantially influence the optical responses of plasmonic sensors, we therefore have performed a detailed numerical analysis of the materials used. We assess the impact of the TiO_2_ /Au/graphene layers, conduct an analysis of the thickness of the Au layer, and conduct an analysis of the graphene layers to find the best sensitivity of the sensor. The numerical results exhibit angular sensitivities of 210 deg/RIU, 245.83 deg/RIU, 264.285 deg/RIU, 285.71 deg/RIU, 292.86 deg/RIU, and 278.57 deg/RIU, respectively, for skin (basal), cervical (HeLa), adrenal gland (PC12), blood (Jurkat), and breast (MCF-7, and MDA-MB-231) cancer types. Additionally, the obtained detection accuracy (DA), figure of merits (FOM), and signal-to-noise ratio (SNR) demonstrate values of 0.263 deg^−1^, 48.02 RIU^−1^, and 3.84, respectively. Moreover, the 2D graphene layer improves the diversity of biosensing applications as well as enhances the biological detection capability of the biosensor by absorption of a biomolecule and bonding with the carbon-based rings that widely exist in biomolecules. For this, we have also analyzed our sensor for the detection of a wide range of biological solutions, obtaining a high sensor linear regression coefficient (R^2^) of 0.9858. As the proposed biosensor could be accomplished utilizing the subsisting fabrication technologies and as enormous advances in nanotechnology have demonstrated significant breakthroughs in plasmonic sensing, this might bring many extensively promising opportunities in future medical applications for cancer detection and other biosensing applications.

## Figures and Tables

**Table 1:** Proposed sensor structural design parameter at the wavelength of 633 nm.

Layer	Material	Refractive Index	Thickness	Ref
1st layer	$\mathrm{Prism}\;({\mathrm{BK}}_{7}$)	1.5151	1.5 μm	[46]
2nd layer	${\mathrm{TiO}}_{2}$	1.99	10 nm	[49]
3rd layer	Gold (Au)	0.13783 + 3.6196 × i	50 nm	[46,53]
4th layer	Graphene (monolayer)	3 + 1.1491 × i	0.34 nm	[46,54,55]
Final layer	Analyte layer	$n_{a}$	1.448	[31,32]

**Table 2:** Normal and cancerous cell analyte refractive index data.

Cancer Type	Cell Specifications	Concentrations	$\mathrm{Cell}\;\mathrm{Refractive}\;\mathrm{Index}\;(n_{a})$
Skin	Healthy basal cell	(30–70%)	1.360
	Cancerous basal cell	80%	1.380
Cervical	Healthy HeLa cell	(30–70%)	1.368
	Cancerous HeLa cell	80%	1.392
Blood	Healthy Jurkat cell	(30–70%)	1.376
	Cancerous Jurkat cell	80%	1.390
Adrenal gland	Healthy PC12 cell	(30–70%)	1.381
	Cancerous PC12 cell	80%	1.395
Breast	Healthy MDA-MB-231 cell	(30–70%)	1.385
	Cancerous MDA-MB-231 cell	80%	1.399
Breast	Healthy MCF-7 cell	(30–70%)	1.387
	Cancerous MCF-7 cell	80%	1.401

**Table 3:** Impact of material layers on the sensitivity of various types of cancer.

Biosensor Structures	Skin Basal Cancer Detection (deg/RIU)	Cervical HeLa Cancer Detection (deg/RIU)	Blood Jurkat Cancer Detection (deg/RIU)	Adrenal Gland PC12 Cancer Detection (deg/RIU)	Breast MDA-MB-231 Cancer Detection (deg/RIU)	Breast MCF-7 Cancer Detection (deg/RIU)
Only Au layer (BK_7_/Au(50 nm)/analyte)	195	229.1667	242.8571	257.1429	292.8571	307.1429
Only Au/Graphene layer (BK_7_/Au(50 nm)/graphene/analyte)	205.0000	237.5000	250.0000	271.4286	285.7143	285.7143
**Proposed TiO_2_/Au/graphene (BK_7_/TiO_2_/Au(50 nm)/graphene/analyte)**	**210.0000**	**245.833**	**264.2857**	**285.7143**	**292.8571**	**278.57**

**Table 4:** Analysis of gold (Au) layer thickness on the angular sensitivity of the proposed BK_7_/TiO_2_/Au/graphene/analyte biosensor for cancer detection.

Biosensor Structure Gold (Au) Thickness	Skin Basal Cancer Detection (deg/RIU)	Cervical HeLa Cancer Detection (deg/RIU)	Blood Jurkat Cancer Detection (deg/RIU)	Adrenal Gland PC12 Cancer Detection (deg/RIU)	Breast MDA-MB-231 Cancer Detection (deg/RIU)	Breast MCF-7 Cancer Detection (deg/RIU)
45 nm	200.0000	233.3333	250.0000	271.4286	285.7143	292.8571
**50 nm**	**210.0000**	**245.8333**	**264.2857**	**285.7143**	**292.8571**	**278.57**
55 nm	215.0000	254.1667	264.2857	285.7143	271.4286	250.0000

**Table 5:** Analysis of graphene layers on the angular sensitivity of the proposed BK_7_/TiO_2_/Au (50 nm)/graphene/analyte biosensor for cancer detection.

Biosensor Structure Graphene Layer No. (L)	Skin Basal Cancer Detection (deg/RIU)	Cervical HeLa Cancer Detection (deg/RIU)	Blood Jurkat Cancer Detection (deg/RIU)	Adrenal Gland PC12 Cancer Detection (deg/RIU)	Breast MDA-MB-231 Cancer Detection (deg/RIU)	Breast MCF-7 Cancer Detection (deg/RIU)
**1**	**210.0000**	**245.8333**	**264.2857**	**285.7143**	**292.8571**	**278.57**
2	220.0000	250.0000	257.1429	264.2857	242.8571	207.1429
3	220.0000	237.5000	250.0000	221.4286	178.5714	142.8571

**Table 6:** Sensitivity analysis of the proposed biosensor for healthy and cancerous cell analyte detection.

Cancer Type	Cell Name	Refractive Index Change (Δn)	SPR $\mathrm{Angle}\;(\theta_{SPR})$	$\mathrm{SPR}\;\mathrm{Angle}\;\mathrm{Shift}\;(\Delta \theta_{spr})$	Reflectance Intensity (%)	Reflectance Intensity Change (%)	Sensitivity $(\Delta \theta_{spr}$/ Δn) (deg/RIU)
Skin	Healthy basal cell	Ref	75.6	Ref	5.684	Ref	Ref
	Cancerous basal cell	0.02	79.8	4.2	12.17	6.486	210
Cervical	Healthy HeLa cell	Ref	77.1	Ref	7.636	Ref	Ref
	Cancerous HeLa cell	0.024	83	5.9	25.08	17.44	245.83
Blood	Healthy Jurkat cell	Ref	78.8	Ref	10.29	Ref	Ref
	Cancerous Jurkat cell	0.014	82.5	3.7	21.66	11.37	264.2857
Adrenal gland	Healthy PC12 cell	Ref	80	Ref	12.75	Ref	Ref
	Cancerous PC12 cell	0.014	84	4	31.79	19.04	285.7143
Breast	Healthy MDA-MB-231 cell	Ref	81	Ref	15.72	Ref	Ref
	Cancerous MDA-MB-231 cell	0.014	85.1	4.1	44.35	28.63	292.857
Breast	Healthy MCF-7 cell	Ref	81.6	Ref	17.71	Ref	Ref
	Cancerous MCF-7 cell	0.014	85.4	3.9	52.06	34.35	278.57

**Table 7:** Comparison of the proposed biosensor with some recently reported works.

Reference	Reported Year	Model Structures	Angular Sensitivity $\left(deg/RIU\right)$	DA (Deg^−1^)	FOM (RIU^−1^)	SNR
[84]	2020	SF_11_/Au/MoS_2_/graphene	130	-	17.02	-
[85]	2020	SF_11_/Au/MoS_2_/WS_2_/WSe_2_	142	-	-	-
[86]	2020	BK_7_/Au/WSe_2_/graphene	178.87	-	27.86	-
[46]	2020	Prism/Ag/PtSe_2_/WS_2_	194	-	17.64	-
[71]	2022	BK_7_/Au/WSe_2_/PtSe_2_/BP	200	0.088	17.70	-
[87]	2021	BK_7_/ZnO/Si/MXene/sensing	231	0.17	-	-
[69]	2019	Trilayers of graphene	121.67	-	-	2.21
[69]	2019	Six MoS_2_ and mono graphene	200	-	-	0.7692
[88]	2021	MoS_2_-graphene hybrid	130	-	-	1.37
[89]	2022	BK_7_/Au/GeS	260	0.143	33.4	-
[85]	2022	Bk_7_ Prism/Ti/Ag/MoS_2_/graphene	144.72	-	-	-
[86]	2022	SiO_2_/Au/Ga-doped ZnO/MXene	264.59	0.115	30.48	-
Our work	2022	BK_7_/TiO_2_/Au/graphene	292.857	0.263	48.02	3.84

**Table 8:** Different existing measurement methods and sensitivity definitions.

Measured Magnitude	Sensitivity Definition	Enhancement Mechanism	Wavelength (nm)	Sensitivity	Ref
Reflected Intensity	$\frac{\mathrm{dR}}{{\mathrm{dn}}_{a}}$ (1/RIU)	LSPR cytop/Au/TMDCs	633	500	[92]
		MoS_2_-based	1540	970	[93]
		Graphene/Ag	1000	455	[94]
Resonance Wavelength	$\frac{{d\lambda }_{\mathrm{spr}}}{{\mathrm{dn}}_{a}}$ (nm/RIU)	Gold on SF11	700	2750	[95]
		Long-range SPR	700	30,000	[96]
		Long-range SPR optimized	830	570,000	[97]
Resonance Angle	$\frac{{d\theta }_{\mathrm{spr}}}{{\mathrm{dn}}_{a}}$ (deg/RIU)	TiO_2_/Au/graphene	633	292.857	Our model

## Data Availability

Not applicable.
